# Preservation of swallowing in resected oral cavity squamous cell carcinoma: examining radiation volume effects (PRESERVE): study protocol for a randomized phase II trial

**DOI:** 10.1186/s13014-020-01636-x

**Published:** 2020-08-14

**Authors:** Pencilla Lang, Jessika Contreras, Noah Kalman, Claire Paterson, Houda Bahig, Astrid Billfalk-Kelly, Sinead Brennan, Kathy Rock, Nancy Read, Varagur Venkatesan, Jinka Sathya, Lucas C. Mendez, S. Danielle MacNeil, Anthony C. Nichols, Kevin Fung, Adrian Mendez, Eric Winquist, Sara Kuruvilla, Paul Stewart, Andrew Warner, Sylvia Mitchell, Julie A. Theurer, David A. Palma

**Affiliations:** 1 Division of Radiation Oncology, London Health Sciences Centre, 800 Commissioners Rd. E, London, ON N6A 5W9 Canada; 2 Department of Radiation Oncology, Miami Cancer Institute, Baptist Health South Florida, Miami, Florida USA; 3 Beatson West of Scotland Cancer Centre, Glasgow, UK; 4 Department of Radiation Oncology, Centre Hospitalier de l’Université de Montréal, Montreal, Quebec Canada; 5 Department of Radiation Oncology, Eastern Health, St. John’s, Newfoundland Canada; 6 Saint Luke’s Radiation Oncology Network, Dublin, Ireland; 7 Department of Radiation Oncology, Cork University Hospital, Cork, Ireland; 8 Department of Otolaryngology – Head and Neck Surgery, Western University, London, Ontario Canada; 9 Department of Medical Oncology, Western University, London, Ontario Canada; 10 School of Communication Sciences and Disorders, Western University, London, Ontario Canada

**Keywords:** Head and neck cancer, Oral cavity, Radiotherapy, Recurrence, Survival, Quality of life, Randomized controlled trial, De-escalation

## Abstract

**Background:**

Patients with resected oral cavity squamous cell carcinoma (OCSCC) are often treated with adjuvant radiation (RT) ± concomitant chemotherapy based on pathological findings. Standard RT volumes include all surgically dissected areas, including the tumour bed and dissected neck. RT has significant acute and long-term toxicities including odynophagia, dysphagia, dermatitis and fibrosis.

The goal of this study is to assess the rate of regional failure with omission of radiation to the surgically dissected pathologically node negative (pN0) hemi-neck(s) compared to historical control, and to compare oncologic outcomes, toxicity, and quality of life (QoL) profiles between standard RT volumes and omission of RT to the pN0 neck.

**Methods:**

This is a multicentre phase II study randomizing 90 patients with T1–4 N0–2 OCSCC with at least one pN0 hemi-neck in a 1:2 ratio between standard RT volumes and omission of RT to the pN0 hemi-neck(s). Patients will be stratified based on overall nodal status (nodal involvement vs. no nodal involvement) and use of concurrent chemotherapy. The primary endpoint is regional failure in the pN0 hemi-neck(s); we hypothesize that a 2-year regional recurrence of 20% or less will be achieved. Secondary endpoints include overall and progression-free survival, local recurrence, rate of salvage therapy, toxicity and QoL.

**Discussion:**

This study will provide an assessment of omission of RT to the dissected pN0 hemi-neck(s) on oncologic outcomes, QoL and toxicity. Results will inform the design of future definitive phase III trials.

**Trial registration:**

Clinicaltrials.gov identifier: NCT03997643. Date of registration: June 25, 2019, Current version: 2.0 on July 11 2020.

## Background

Patients with resected squamous cell carcinoma of the oral cavity (OCSCC) are at risk of locoregional failure recurrence (LRR), at either the tumour surgical bed or in the neck. Postoperative radiotherapy (PORT) is often added after surgical resection to reduce the risk of local and regional recurrence in patients with high-risk features. Guidelines generally recommend PORT in patients with more than one lymph node involved, pT3 or pT4 primary disease, lymphovascular invasion (LVI), perineural invasion (PNI), close or positive margins, extranodal extension (ENE), and sometimes for patients with lymph node involvement of neck levels IV or V [1–4].

ENE and positive surgical margins are considered the highest-risk pathological features, whereas the other adverse features are considered ‘intermediate risk’. Patients with high-risk features are generally offered concurrent chemotherapy with PORT, based on a post-hoc analysis two of randomized trials [5–7]. In patients with other adverse features, there is limited randomized evidence of the benefits of PORT alone [8–10]. Retrospective series and comparisons with historical controls have shown reductions in LRR and improvement in overall survival (OS) with PORT [11–18].

RT treatment volumes after surgery generally include the entire surgical bed, including the dissected hemi-neck(s), and may include the contralateral undissected neck at the discretion of the treating physician [19]. However, PORT is associated with significant acute and late toxicities, including dysphagia, mucositis, xerostomia, dermatitis, fibrosis, osteoradionecrosis, voice changes, ototoxicity and hypothyroidism [7, 20]. Larger RT treatment volumes are associated with increasing toxicity. These large treatment volumes are based on historical practice, without guidance from randomized evidence. The benefit of treating the nodal regions in the pathologically node negative (pN0) neck is unknown.

Retrospective studies looking at series of oral cavity cancer patients in whom PORT was omitted altogether have also demonstrated low rates of isolated nodal recurrence ranging from 8 to 15% [21–23]. In their series of 166 patients with mostly T1-T2 oral tongue tumours with a pN0 neck treated with surgery alone (75%), PORT to the primary site (8%) or PORT to the primary site and neck (17%), So et al. reported a rate of isolated nodal recurrence of 7–8% in all groups [21]. Mizrachi et al. described a series of 558 patients with T1-T4 disease and pN0 neck with 65% not receiving PORT, with a neck recurrence rate of 10% [22]. In Ganly et al., 164 patients with pT1-T2 pN0 oral tongue cancer without PORT had an isolated regional recurrence rate of 15%, with an additional 5% having a simultaneous locoregional recurrence. In this study depth of invasion was predictive of neck recurrence [23]. In a series of 127 patients with a pN1 neck, the neck recurrence-free survival was 85% in those receiving PORT vs 84% in those without PORT [24]. It is difficult to draw comparisons between groups receiving or not receiving PORT in these retrospective studies since those receiving PORT had more risk features.

Retrospective data from a small number of PORT volume studies also demonstrate good oncologic outcomes in patients where radiation was omitted to the contralateral clinically or pathologically N0 neck [25, 26]. In Vegeer et al. 123 patients with well-lateralized oral cavity or oropharynx squamous cell carcinoma (SCC) were treated with unilateral PORT, with 60% of patients N0 and 41% N1 or N2. Contralateral metastases developed in only 6% of patients, with most successfully receiving salvage therapy [25].

A recent non-randomized prospective phase II trial eliminated PORT to the pN0 neck in 72 patients with head and neck squamous cell carcinoma (HNSCC), demonstrating excellent results with no isolated failures and 97% control in the unirradiated pN0 neck. Twenty percent of patients included in the study had oral cavity tumours [27].

Taken together, the existing retrospective and prospective data suggests that omitting PORT in patients with a pN0 neck likely has a recurrence rate less than 15–20%. However, no randomized studies have directly examined the omission of PORT in the pN0 neck. There is also a paucity of data examining the effects of omission of PORT on QoL. We hypothesize that omitting PORT to the hemi-neck(s) that have been dissected and shown to be pN0 will be associated with acceptable rates of regional recurrence and will improve quality of life (QoL). The goal of this randomized phase II study is to assess oncologic outcomes, functional outcomes, and QoL in patients treated with PORT to the historically standard volumes (usually including the primary site and all dissected neck areas) vs. PORT only to the primary site and pathologically involved hemi-neck, omitting radiation to the pN0 hemi-neck(s).

## Methods / design

### Objectives

The objectives of this trial are to:

1. Compare regional recurrence rate to historical controls with omission of PORT to the pN0 neck.
2. Compare oncologic outcomes, toxicity and QoL for PORT (± chemotherapy) based on standard treatment volumes (including the primary site tumour bed, dissected neck ± elective nodal regions) vs. PORT [± chemotherapy] that avoids treating the dissected, pN0 neck.

It is generally accepted that a risk of nodal recurrence of 15–20% is sufficient to warrant radiation to a nodal basin. Our hypothesis is that for patients with SCC of the oral cavity, T1–4 N0–3 (as per AJCC 8th edition) with at least one surgically dissected pN0 hemi-neck, the regional failure rate will be 20% or less at 2 years when treated with PORT omitting the pN0 neck.

### Study design

**Fig. 1:**
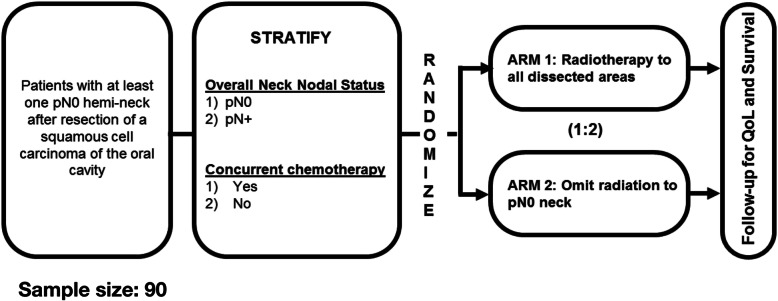
Study schema

This is an open-label phase II multi-centre randomized trial. Patients will be randomized between current standard of care treatment (Arm 1) vs. omission of radiation to the pN0 dissected hemi-neck(s) (Arm 2) in a 1:2 ratio (Fig. 1). The required sample size is 90 patients. Stratification factors include neck nodal status (pN0 vs pN+) and use of chemotherapy. Patients will be recruited from tertiary care centres (full list of participating sites available on clinicaltrials.gov, NCT03997643).

#### Primary endpoint

- Regional failure in the pN0 hemi-neck(s)

#### Secondary endpoints

- OS
- Disease-free survival (DFS)
- Local recurrence
- Regional recurrence
- Locoregional recurrence
- Rate of salvage treatment (surgery ± radiotherapy) in the pN0 neck, and freedom from unsalvageable neck recurrence
- QoL at 1 year, assessed with the MD Anderson Dysphagia Inventory (MDADI) [28], the European Organisation for Research and Treatment of Cancer (EORTC) Quality of Life Cancer Patients general (QLQ-C30) [29] and head & neck (H&N35) scales [30], the EuroQOL 5-Dimension 5-Level (EQ-5D-5L) [31], and the Neck Dissection Impairment Index (NDII) [32]
- QoL measured at treatment completion and 6, 12, 15,18, 24, 30, 36, 42, 48, 54 and 60 months, measured with MDADI, EORTC QLQ-C30, H&N35, EQ-5D-5L and NDII
- Rate of feeding tube insertion after start of radiation (either gastric, gastrojejeunal, or nasogastric) and rate of feeding tube use at 1-year post-randomization.
- Swallowing function at 1-year, assessed by Modified Barium Swallow (MBS) study and measured by the Modified Barium Swallow Impairment Profile (MBSImP™©) score [33], the Dynamic Imaging Grade of Swallowing Toxicity (DIGEST™) score [34], and the Functional Oral Intake Scale (FOIS) [35].
- Toxicity, assessed using the National Cancer Institute Common Toxicity Criteria (NCI-CTC) version 4.03
- Rate of failure in the clinically node negative neck, if applicable (i.e. the undissected node-negative neck, for well-lateralized tumours).

#### Inclusion criteria

- Age 18 years or older
- Willing to provide informed consent
- Eastern Cooperative Oncology Group (ECOG) performance status 0–2
- Resected OCSCC with at least an ipsilateral selective neck dissection. The oral cavity includes: lips, buccal mucosa, oral tongue, floor of mouth, gingiva, retromolar trigone, and hard palate
- Patient has at least one pathological feature that is an indication for PORT: positive or close (≤ 3 mm) margin, presence of LVI or PNI, pT3 or pT4 disease, positive lymph nodes, or ENE
- PORT is recommended by the treating physician
- Pathologically lymph node negative in at least one dissected hemi-neck with at least 10 nodes recovered in each pN0 hemi-neck, after a dissection that at minimum includes nodal levels 1–3 in the pN0 hemi-neck(s).
- Radiation contours have been peer-reviewed and approved

#### Exclusion criteria

- Patients with an ipsilateral neck dissection only with positive lymph nodes, unless they undergo a contralateral neck dissection that is pN0
- Patients with bilaterally involved neck nodes
- Patients with pT3-T4 tumours **involving midline** who undergo an ipsilateral neck dissection (unless a contralateral neck dissection is performed)
- Serious medical comorbidities or other contraindications to radiotherapy
- Prior history of head and neck cancer within 5 years
- Any other active invasive malignancy, except non-melanotic skin cancers, low-risk prostate cancer, and Stage I-IVA papillary or follicular thyroid cancer.
- Prior head and neck radiation at any time
- Prior oncologic head and neck surgery in the oral cavity or neck.
- Known metastatic disease
- Locoregional disease recurrence identified following surgical resection but prior to start of radiotherapy
- Inability to attend full course of radiotherapy or follow-up visits
- Unable or unwilling to complete QoL questionnaires
- Pregnant or lactating women

#### Pre-treatment evaluation

- History and physical examination by a radiation oncologist within 5 weeks prior to randomization
- Staging prior to randomization:
  - ◦ CT or MRI of the neck (with contrast unless contraindicated) within 14 weeks of randomization. This can include the pre-operative CT, or the radiation therapy CT or MRI simulation if reviewed by a radiologist.
    - ▪ In some instances, suspicious lymph nodes are visible on the scan after surgery. In such instances, recurrence must be ruled out pathologically before enrollment, either with a needle biopsy or resection of these nodes.
  - ◦ CT of the chest or whole body PET-CT (usually prior to surgery, must be within 14 weeks of randomization)
- Histological confirmation of SCC
- Pregnancy test for women of child-bearing age within 2 weeks of randomization
- Dental evaluation prior to starting treatment, except edentulous patients
- Assessment of dysphagia using NCI-CTC version 4.03 within 2 weeks of treatment initiation
- Completion of QoL scoring prior to initiation of treatment
- Prior to randomization, radiation contours are to be peer-reviewed and approved
- MBS at baseline prior to initiation of treatment with documentation of the MBSIimP™©, DIGEST™ and the FOIS scores

#### Treatment plan

Surgical resection, and adjuvant radiotherapy and chemotherapy will be delivered in accordance with National Comprehensive Cancer Network (NCCN) Clinical Guidelines [1].

Primary tumours should be resected en bloc whenever possible with the goal of achieving clear margins. Patients with midline involvement of the primary tumour should receive a bilateral neck dissection.

Adjuvant cisplatin-based chemotherapy concurrent with radiotherapy is at the discretion of the treating medical oncologist, and is recommended for patients with positive margins or ENE (for patients who can tolerate chemotherapy). For patients who are deemed unfit or too elderly (> 70 years of age) for cisplatin-based chemotherapy, the standard dose and/or schedule can be modified, alternative systemic therapy regimens maybe used (eg. weekly carboplatin, Calais regimen), or systemic therapy can be omitted at the discretion of the treating physicians. It is strongly recommended that radiation starts within 6 weeks of the date of surgery, and it is mandatory to start no more than 7 weeks after the date of surgery.

During treatment, supportive care should be in accordance with local standard of care, which often includes speech language pathology (SLP) assessment. Any SLP interventions (e.g. providing swallowing exercises) should be the same in both arms and conform to local standard of care.

##### Dose/fractionation

In both arms, a dose of 60 Gy in 30 fractions will be delivered to the operative bed target volumes. Centres that normally treat dissected, node-negative levels to 54 Gy in 30 fractions will be permitted to do so, if used consistently for all patients on trial. Areas of positive margins or ENE should receive 64 Gy in 30 fractions if those areas can be localized. Undissected areas that require coverage, in the opinion of the treating radiation oncologist (e.g. low neck, retrostyloid space), should receive 54 Gy in 30 fractions (Table 1).

**Table 1:** Radiation treatment volumes and doses

PTV Volume	CTV Volumes Included		Dose in 30 fractions
	Arm 1	Arm 2	
**PTV64** Areas of positive margin or ENE	CTV64	CTV64	64 Gy
**PTV60** Dissected neck	CTVp60 CTVn60pos CTVn60neg	CTVp60 CTVn60pos	60 Gy
**PTV54** **(optional)** Not surgically dissected elective nodal regions	CTVn54pos CTVn54neg	CTVn54pos	54 Gy

##### Immobilization and localization

All patients will be immobilized in a custom thermoplastic shell and will undergo a planning CT simulation (with or without IV contrast) encompassing the head and neck to below the clavicles. The planning CT will be fused with other diagnostic imaging (e.g. MRI scans or pre-operative CT) where necessary. Bite blocks / tongue depressors / jaw separators may be used as per institutional protocol; these must be determined at the time of planning CT, prior to contour generation and patient randomization.

##### Radiotherapy volume definitions

**Fig. 2:**
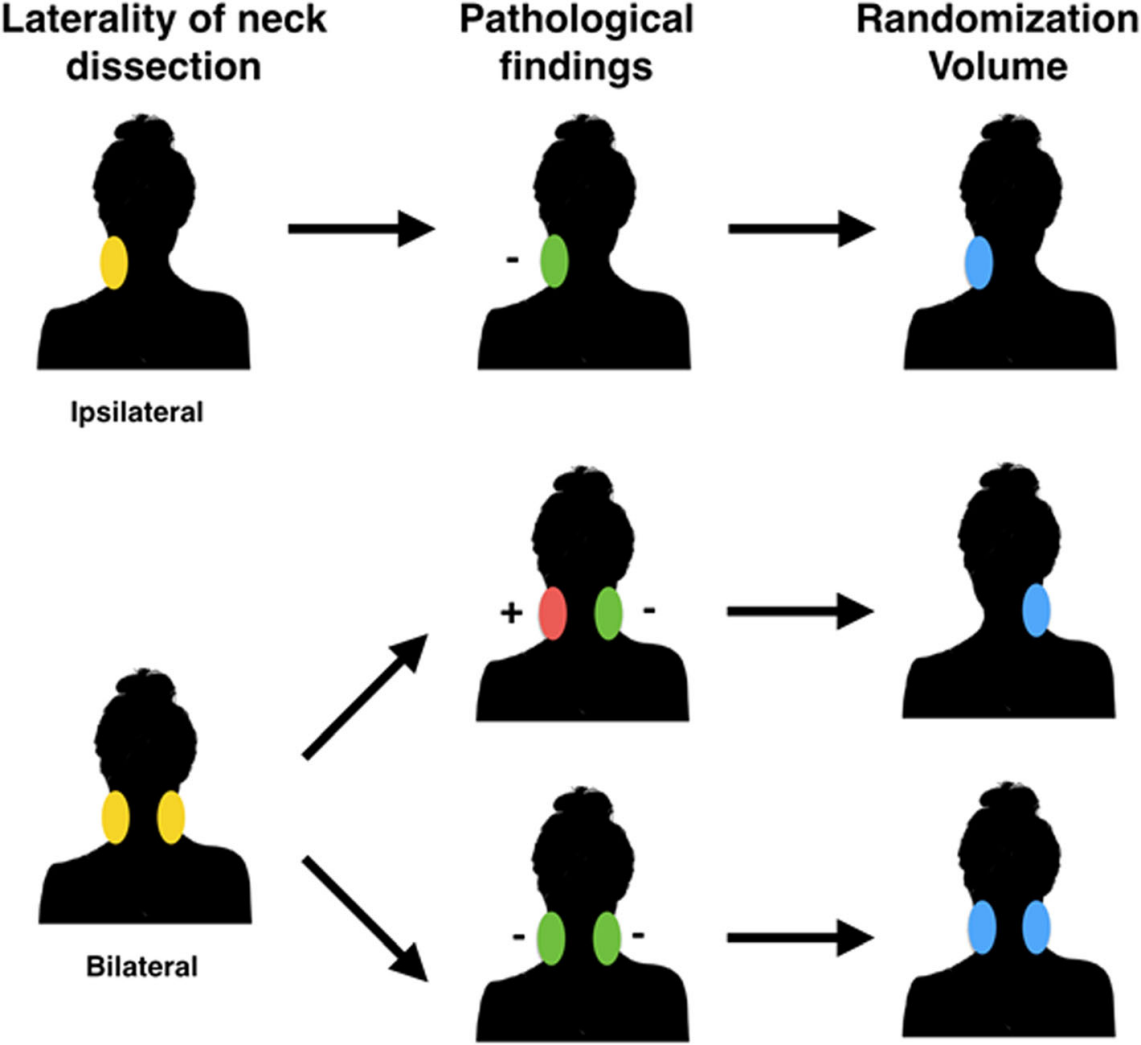
The “Randomization Volume” corresponds to the pN0 hemi-neck(s). The neck volumes included in the “Randomization Volume” depend on whether the patient had an ipsilateral vs. bilateral neck dissection, and the pathological findings in each hemi-neck(s). Patients with bilaterally involved neck nodes are ineligible. Patients with an ipsilateral neck dissection with positive lymph nodes are ineligible unless they undergo a contralateral neck dissection that is pN0. If randomized to standard treatment volumes (Arm 1), all contoured volumes, including the “Randomization Volume” will be treated. If randomized to omission of the pN0 neck (Arm 2), the “Randomization Volume” will be omitted from treatment planning

A randomization volume will be defined as the nodal volume in the dissected pN0 hemi-neck(s). The randomization volume will depend on the laterality of the neck dissection performed (ipsilateral vs. bilateral), and whether pathologically involved nodes are present in the neck dissection, defined in Fig. 2. Patients with bilaterally involved neck nodes are ineligible. Patients with a unilateral neck dissection with positive lymph nodes are ineligible unless they undergo a staged neck dissection of the opposite side that is pN0.

**Standard treatment volumes (Arm 1):** If randomized to Arm 1, all contoured volumes, including the randomization volume will be treated.

**Omission of pN0 neck (Arm 2):** If randomized to Arm 2 the randomization volume will be omitted from treatment planning.

**Clinical Target Volumes (CTV):**

The following radiation volumes will be contoured for all patients prior to randomization (Table 1). The suffix “pos” denotes CTVs in the node-positive hemi-neck, and the suffix “neg” denotes the CTVs in the node-negative hemi-neck.

1. **High-risk volume (CTV64):** regions corresponding to positive margins or ENE, if present, and if those areas can be localized.
2. **Primary tumour operative bed (CTVp60):** surgically dissected areas corresponding to the resected primary tumour, typically including the pre-operative tumour area and any flap reconstructions and clips with a margin.
3. **Involved neck:**
  - **Surgically dissected involved neck nodal volume (CTVn60pos):** surgically dissected neck levels in the node-positive hemi-neck, if applicable. In the dissected neck, some centres routinely treat only involved nodal areas to 60 Gy and the remainder of the dissected neck to 54 Gy. For centres where this is standard, this approach is acceptable, but all patients enrolled from these centres must be treated with this approach. The areas treated to 54 Gy would be included in a CTVn54pos.
  - **Optional uninvolved low-risk neck on the involved side (CTVn54pos):** low-risk undissected neck nodal volume on the involved side, if applicable at the discretion of the radiation oncologist. This may include the standard lymphatic drainage sites not dissected at the time of surgery in the node positive hemi-neck such as the nodal levels below or above the dissected areas (e.g. level IVb, retrostyloid space).
4. **“Randomization Volume” corresponding to the Uninvolved neck:**
  - **Surgically dissected involved neck nodal volume (CTVn60neg):** surgically dissected neck levels in the node negative hemi-neck. In centres that routinely treat the pN0 neck to 54 Gy, that dose is acceptable, but all patients must be treated with that approach. In such centres, these areas treated to 54 Gy would be included in a CTVn54neg.
  - **Optional uninvolved low-risk neck on the uninvolved side (CTVn54neg):** low-risk undissected neck nodal volume on the uninvolved side, if applicable at the discretion of the radiation oncologist. In a patient who has had a unilateral neck dissection that was pN0, this may include the contralateral clinically node negative (cN0) neck if that is standard institutional practice

**Fig. 3:**
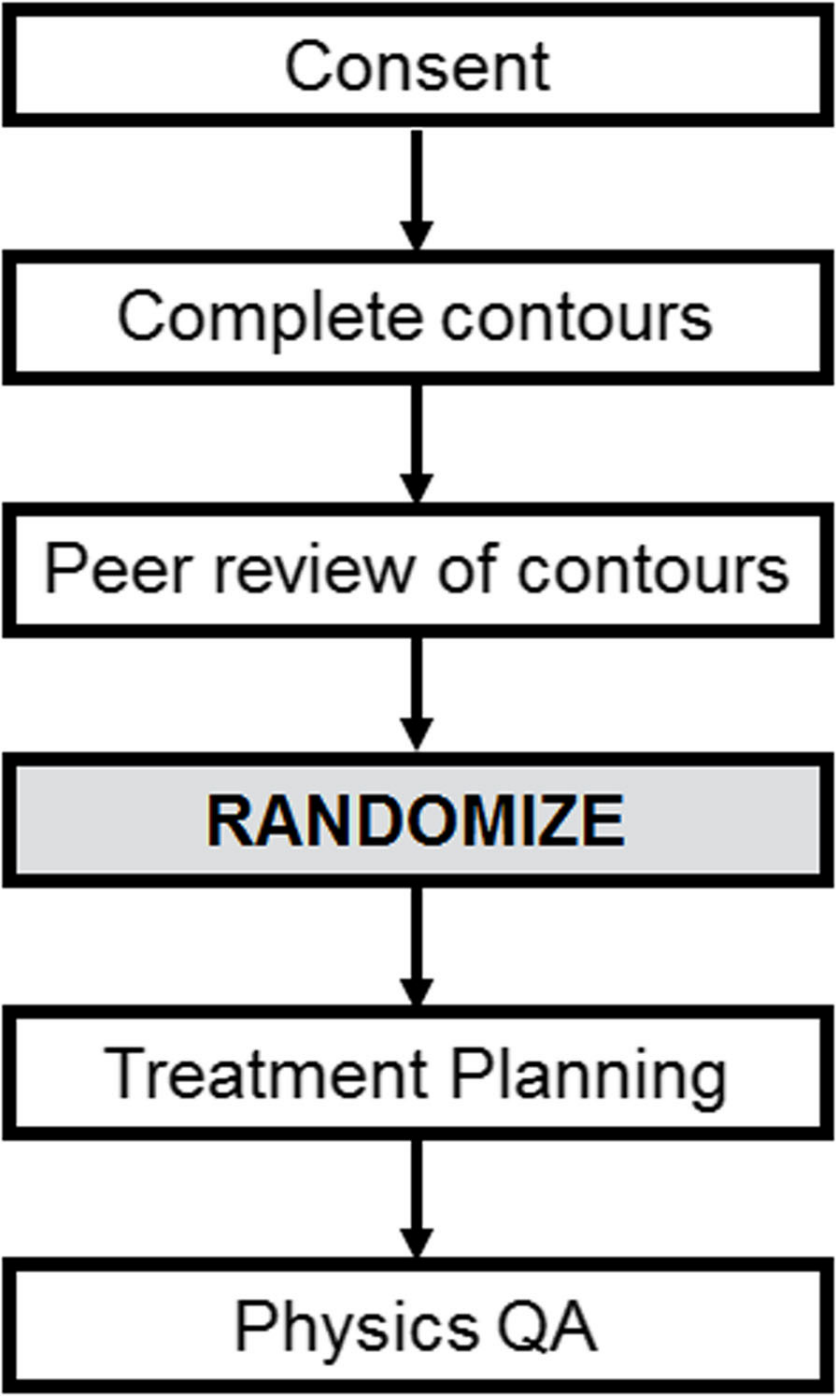
Flowchart showing timing of randomization with respect to peer review and treatment planning. Contours must be finalized before randomization and may not be changed after randomization

If randomized to omission of PORT to pN0 neck (Arm 2), the CTVn60neg and CTV54neg contours are deleted after randomization, prior to treatment planning. To prevent bias, contours cannot be changed after randomization. Local peer review of contours must take place before randomization occurs. An overview of the protocol timeline is shown in Fig. 3.

**Planning Target Volumes (PTV):**

A 3–5 mm expansion is used around the combined CTVs (Table 1) to create the PTV, as per institutional setup and protocol.

##### Radiotherapy planning

Intensity modulated radiotherapy (IMRT) photon therapy or proton therapy will be used for all patients in this study. IMRT can be delivered using static-beam techniques or rotational techniques (e.g. Tomotherapy or Volumetric Modulated Arc Therapy [VMAT]). If protons are used the dose will be reported in Gy (relative biological effectiveness [RBE]) where the proton dose is multiplied by an RBE of 1.1. All reported doses in Gy are considered equivalent. Centres with proton therapy will use their institutional standard planning and delivery techniques.

All plans will be normalized to ensure that 95% of each PTV is covered by 95% of the prescription dose for that volume. An exception will be allowed for centres that normally prescribe in such a manner that 95% of the PTV be covered by 100% of the prescription dose, and such centres must pre-specify this before enrolling the first patient, and all subsequent patients must be planned in the same manner. A modified PTV cropped 3-5 mm from the external contour for dose evaluation may be used as per institutional guidelines.

The maximum dose to PTV64 (or PTV60 if no PTV64 present) should not exceed 110% of the prescribed dose, and no volume > 1 cc outside of these PTVs should receive > 105% of the prescription dose.

Organs at Risk (OAR) definitions, dose constraints and planning priorities are adapted from the following protocols: RTOG protocols 1016 [36] (Arm 1) and RTOG 0920 [37] (Arm 2), NCIC-CTG HN6, ORATOR [38, 39] and ORATOR2 [40], and are described in Additional file 1.

##### Quality assurance (QA)

In order to ensure patient safety and effective treatment delivery, a robust QA protocol is incorporated. The following requirements must be completed for each patient:

- Prior to randomization, each set of contours will be peer-reviewed, either by another individual radiation oncologist or at a team head and neck QA rounds.
- All dose delivery for intensity-modulated plans (including arc-based treatments) will be confirmed before treatment by physics staff.

Cone-beam CT and/or orthogonal x-rays will be used on a daily basis to verify treatment positioning, as per institutional standard practice.

##### Credentialling

Prior to enrolling patients, each centre will be given a sample CT dataset through secure file transfer protocol (FTP) for contouring, planning and physics QA. Enrollment can begin once the plan and QA have been approved at the London Regional Cancer Program. Centres who have been accredited for ORATOR [38] or ORATOR2 [40] are exempt from this requirement.

#### Participant discontinuation / withdrawal

Participants may voluntarily discontinue participation in the study at any time. If a participant is removed from the study, the clinical and laboratory evaluations that would have been performed at the end of the study should be obtained. If a participant is removed because of an adverse event, they should remain under medical observation as long as deemed appropriate by the treating physician.

#### Follow-up evaluation

Day 1 of follow-up will be the first day of radiotherapy. Follow-up will consist of history and physical examination with laryngopharyngoscopy, CT imaging of the neck ± thorax, QoL assessments and MBS. The follow-up schedule is shown in Additional file 2. Additional imaging or laboratory investigations should be carried out at the discretion of the oncologist, based on findings in the history or physical examination. Additional treatment (e.g. salvage treatment with surgery or further radiotherapy) is at the discretion of the treating physicians but will be recorded in the case report form.

#### Measurement of outcomes

- **pN0 neck failure:** measured as time from randomization until disease recurrence in the initially pN0 hemi-neck(s). Patients with prior or simultaneous recurrence at the primary site or in the initially pN+ hemi-neck will be censored for this outcome as of that timepoint. The primary endpoint is a comparison of pN0 neck failure in Arm 2 vs. historical controls; this endpoint will also be compared between the two arms as a secondary endpoint.
- **OS:** measured as time from randomization until death from any cause.
- **DFS:** measured as time from randomization to either recurrence at any location or death, whichever occurs first. New primary tumours will not count as DFS events.
- **Local recurrence:** measured as time from randomization until disease recurrence at the primary site.
- **Regional recurrence:** measured as time from randomization until disease recurrence anywhere in the neck.
- **Locoregional recurrence:** measured as time from randomization until disease recurrence anywhere in the neck or at the primary site, whichever occurs first.
- **Recurrence in the pN0 neck without other locoregional recurrence:** measured as time from randomization until disease recurrence in the pN0 neck alone without recurrence at the primary site or pN+ neck.
- **Rate of salvage surgery and/or radiation in pN0 neck:** measured as time from randomization to salvage intervention (surgery ± radiation) in the pN0 neck. Freedom from unsalvageable neck recurrence will be reported as the time from randomization to development of a neck recurrence in the pN0 neck that could not be salvaged.
- **Feeding Tube Insertion:** Rate of feeding tube insertion after start of radiation (either gastric, gastrojejeunal, or nasogastric) and rate of feeding tube use at 1-year post-randomization. Patients with feeding tubes inserted prior to randomization will be censored for this endpoint.
- **Rate of failure in the clinically node negative neck**: for patients who have unilateral neck dissections, the cN0 neck may be treated with radiation or observed, at the discretion of the treating oncologist (see section 6). This is measured as the time from randomization to failure in the cN0 neck, and will be reported for the whole group of patients who had unilateral dissections, and also stratified by whether radiation was delivered to that area.

#### Enrollment, randomization and allocation

Patients will be enrolled by dedicated clinical trials staff and/or the investigator at each participating institution. Patients will randomized in a 1:2 ratio to standard radiation volumes (Arm 1) vs omission to the pN0 neck (Arm 2). A permuted block design with two stratification factors will used with the size of the blocks known only to the statistician, stratified based upon overall neck nodal status (pN0 vs. pN+) and use of concurrent chemotherapy (yes vs. no). Randomization sequences are generated for each strata separately with a random number generator based on permuted block design. This gets formatted as a CVS file which gets uploaded into REDCap [41].

#### Statistical considerations

##### Sample size

The primary endpoint is the rate of relapse in the pN0 neck (i.e. the regional relapse rate in the pN0 neck) in Arm 2, compared to historical controls. All other endpoints are a comparison between Arm 1 and Arm 2.

It is generally accepted that a risk of nodal recurrence of 15–20% is sufficient to warrant radiation to a nodal basin, and therefore we wish to exclude a risk of regional recurrence in the pN0 neck of 20% at 2-years. Using a one-sided, one-sample binomial test, allowing for 5% dropout, a sample size in Arm 2 of 60 patients provides 83.1% power at a 0.05 significance level to detect a regional control rate in the pN0 neck of 92%, compared to an unacceptable level of 80%.

This sample size (30 in Arm 1 and 60 in Arm 2) also allows for > 80% power to detect a 10-point difference in the total MDADI score at 1 year (a secondary endpoint), assuming the scores are normally distributed with a standard deviation of 15 in each arm. It is generally believed that a 10-point difference in standardized QoL scores represents a clinically significant difference in QoL [42]. It is assumed that the QoL scores will be normally distributed with a standard deviation of 15 in each arm. The MDADI will be calculated as the composite score, but will also be reported for each of the subscales.

#### Analysis plan

Patients will be analyzed in the groups to which they are assigned (intention-to-treat). Oncologic outcomes and OS will be calculated from date of randomization using the Kaplan-Meier method. A one-sided, one-sample binomial test will be used for the primary endpoint as described above. For actuarial comparisons between arms, the stratified log-rank test will be used (stratified by stratification factors). An two-sample T-test will be used to compare QoL scores at 1-year. The percentage of patients in each arm who experience a clinically significant QoL decline on the MDADI (10 points) will also be reported.

MBSImP™© scores will be compared at 1-year, including the total score, and the scores on the oral and pharyngeal subscales, using a two-sample T-test or a Wilcoxon rank sum test as appropriate. DIGEST™ and FOIS scores will be compared using the Chi-square test.

Pre-planned subgroup analysis will occur based on the stratification variables (nodal status [pN0 vs. pN+] and use of concurrent chemotherapy), as well as based on the neck dissection performed (unilateral vs. bilateral), and T-stage (T1–2 vs. T3–4). There will also be a pre-planned analysis based on the extent of nodes harvested in the pN0 neck (< 18 vs. 18 or more), depth of invasion of the primary tumour (< 4 mm vs. 4 mm or more), and photon versus proton treatment.

Multivariable Cox proportional hazards or logistic regression analysis, as appropriate, will be used to determine baseline and pathologic factors predictive of pN0 neck failure, DFS, OS, locoregional recurrence and salvage therapy. For the secondary endpoints involving QoL scales, linear mixed effects models will be used; for the MDADI, the total scores will be compared between the two arms, whereas for the EORTC QLQ-C30 and H&N35 scales, each of the subscales (e.g. pain, swallowing, etc.) will be compared between the two arms.

Rates of grade ≥ 2 toxicity and use of feeding tubes will be compared between arms using the Chi-square or Fisher’s exact test, as appropriate.

Utilities will be calculated from the EQ-5D-5L which will be administered at baseline and at 6 month intervals. Quality adjusted life years (QALYs) will be assessed as the area under the preference-weighted survival curve. Overall costs of each treatment strategy will be abstracted from the available literature. The incremental cost effectiveness ratios (ICERs) between treatment arms will be compared through the standard method of ratio between differences in costs and QALYs. Point estimates for these differences can be derived from multivariable generalized estimating equations (GEE) or generalized linear model (GLM) analyses.

#### Data safety monitoring committee

The Data Safety Monitoring Committee (DSMC), consisting of at least one radiation oncologist and one medical oncologist not involved in the study, will meet every 6 months after study initiation. Toxicity outcomes will be monitored, but since the experimental arm involves smaller radiation volumes, it is extremely unlikely that toxicity would be higher in the experimental arm, and therefore no stopping rules for toxicity are included.

##### Interim analysis

The DSMC will conduct one interim analysis once 45 patients have been accrued and completed the 6-month QoL questionnaires. For this analysis, the DSMC will be blinded to the identity of each treatment arm, but QoL, recurrence in the pN0 neck, OS, and DFS estimates at 2-years will be presented for each arm.

The DSMC will recommend stopping the trial if there is an OS difference that is statistically significant with a threshold of *p* < 0.001 using the stratified log-rank test, based on the Haybittle-Peto stopping rule; this retains an overall alpha of 0.05.

#### Ethical considerations

The Principal Investigator will obtain ethical approval and clinical trial authorization by competent authorities according to local laws and regulations. The World Health Organization (WHO) Trial Registration Data Set is shown in Additional file 4.

##### Institutional review board (IRB) / research ethics board (REB)

The protocol (and any amendments), the informed consent form, and any other written information to be given to subjects will be reviewed and approved by a properly constituted IRB/REB operating in accordance with the current federal regulations (e.g., Canadian Food and Drug Regulations (C.05.001); US Code of Federal Regulations (21CFR part 56)), ICH GCP and local regulatory requirements. A letter to the investigator documenting the date of the approval of the protocol and informed consent form will be obtained from the IRB/REB prior to initiating the study. Any institution opening this study will obtain IRB/REB approval prior to local initiation.

##### Informed consent

The written informed consent form (Additional file 3) to be provided to potential study subjects should be approved by the IRB/REB and adhere to ICH GCP and the ethical principles that have their origin in the Declaration of Helsinki. The investigator is responsible for obtaining written informed consent from each subject, or if the subject is unable to provide informed consent, the subject’s legally acceptable representative, prior to beginning any study procedures and treatment(s). The investigator should inform the subject, or the subject’s legally acceptable representative, of all aspects of the study, including the potential risks and benefits involved. The subject should be given ample time and opportunity to ask questions prior to deciding about participating in the study and be informed that participation in the study is voluntary and that they are completely free to refuse to enter the study or to withdraw from it at any time, for any reason.

Informed consent documentation must be signed and dated by the subject, or the subject’s legally acceptable representative, and by the individual who conducted the informed consent discussion. A copy of the signed and dated written informed consent form should be given to the subject or the subject’s legally acceptable representative. The process of obtaining informed consent should be documented in the patient source documents.

##### Confidentiality of subject records

The names and personal information of study participants will be held in strict confidence. All study records (case report forms [CRFs], safety reports, correspondence, etc.) will only identify the subject by initials and the assigned study identification number. The data coordinator will maintain a confidential subject identification list (Master List) during the course of the study. Access to confidential information (i.e., source documents and patient records) is only permitted for direct subject management and for those involved in monitoring the conduct of the study (i.e., sponsors, representatives of the IRB/REB, and regulatory agencies). The subject’s name will not be used in any public report of the study.

##### Data storage

All data will be stored on REDCap [41] a secure web application for building and managing online databases commonly used in the clinical trials research community. Ongoing auditing will be performed by the London Regional Cancer Program (LRCP) clinical trials unit, independent from the trial investigators and sponsor.

##### Adverse events

The severity of adverse events will be evaluated using the NCI-AE) version 4.03 grading scale [43]. Any grade 4 or 5 adverse event that is definitely, probably, or possibly the result of protocol treatment must be reported to the Principal Investigator and Central Office within 24 h of discovery. The Serious Adverse Event (SAE) report form is to be completed with all available information and uploaded to the REDCap SAE page. The Central Office must be notified by email or telephone that a new SAE form has been uploaded into REDCap. It is the responsibility of each local Principal Investigator to report all SAEs to their REB as per local REB requirements. The Principal Investigator should also comply with the applicable regulatory requirement(s) related to the reporting of unexpected serious adverse drug reactions to the regulatory authority(ies).

##### Protocol amendments and trial publication

Any modifications to the trial protocol must be approved and enacted by the Principal Investigator (current version: 2.0 on July 11, 2020). Protocol amendments will be communicated to all participating centres, investigators, IRBs, and trial registries by the Principal Investigator. Any communication or publication of trial results will be led by the Principal Investigator, and is expected to occur within 1 year of the primary analysis. Trial results will remain embargoed until conference presentation of an abstract or until information release is authorized. Authorship of the trial abstract and ultimately the full manuscript will be decided by the Principal Investigator at the time of submission. Professional writers will not be used for either abstract or manuscript preparation.

## Discussion

Patients with OCSCC at increased risk of recurrence are often treated with PORT (± chemotherapy). Current radiation volume recommendations include treatment of the entire surgically dissected area, including the primary tumour bed and the dissected neck(s). Limited prospective and retrospective evidence suggests that the risk of recurrence in the clinically or pathologically negative neck not treated with PORT is low [22, 25, 44].

PRESERVE is a phase II multi-centre trial that aims to randomize 90 patients between standard treatment volumes (Arm 1) and omission of radiation to the pathologically node negative dissected hemi-neck(s). The trial will assess the safety of omitting the pN0 neck from the treatment volume by comparing the regional recurrence rate to historical controls, and will allow for comparisons of regional recurrence, OS, and QoL between the two interventions. To date, no randomized studies exist examining omission of radiation to the uninvolved neck. Results of PRESERVE are expected to provide data that will further inform future phase III randomized trials, and has the potential to guide improvements in QoL for patients with OCSCC worldwide.

## Supplementary information

**Additional file 1.** Dose constraints, OAR definitions, planning priorities.

**Additional file 2.** Follow-up evaluation schedule.

**Additional file 3.** Study information and consent form.

**Additional file 4.** WHO trial registration data set.

## Data Availability

The datasets used and/or analysed during the current study are available from the corresponding author on reasonable request.

## Abbreviations

AJCC: American Joint Committee on Cancer
cN0: Clinically node negative
CRF: Case report form
CT: Computed tomography
CTC-AE: Common Toxicity Criteria for Adverse Events
CTV: Clinical target volume
DIGEST™: Dynamic Imaging Grade of Swallowing Toxicity
DFS: Disease-free survival
DSMC: Data Safety Monitoring Committee
ECOG: Eastern Cooperative Oncology Group
ENE: Extranodal extension
EORTC: European Platform of Cancer Research
EQ-5D-5L: EuroQoL 5 dimensions, 5 levels
FOIS: Functional Oral Intake Score
FTP: File transfer protocol
GEE: Generalized estimating equations
GLM: General linear model
H&N35: European Organisation for Research and Treatment Quality of Life Questionaire, Head and neck module
HNSCC: Head and neck squamous cell carcinoma
ICER: Incremental cost effectiveness ratio
IMRT: Intensity modulated radiotherapy
IRB: Institutional Review Board
VMAT: Volumetric Modulated Arc Therapy
LRCP: London Regional Cancer Program
LRR: Locoregional recurrence
LVI: Lymphovascular invasion
MBS: Modified barium swallow
MBSImP™©: Modified Barium Swallow Impairment
MDADI: MD Anderson Dysphagia Inventory
MRI: Magnetic resonance imaging
NCCN: National Comrephensive Cancer Network
NCI-CTC: National Cancer Institute Common Toxicity Criteria
NDII: Neck Dissection Impairment Index
OAR: Organ at risk
OCSCC: Oral cavity squamous cell carcinoma
OS: Overall survival
PET-CT: 18F-fludeoxyglucose positron emission tomography
PFS: Progression-free survival
pN+: Pathologically node positive
pN0: Pathologically node negative
PNI: Perineural invasion
PORT: Post-operative radiotherapy
PTV: Planning target volume
QA: Quality assurance
QALY: Quality adjusted life year
QLQ-C30: European Organization for Research and Treatment of Cancer Quality of Life Questionnaire, Core Module
QoL: Quality of life
RBE: Relative biological effectiveness
REB: Research ethics board
RT: Radiotherapy
SAE: Serious adverse event
SCC: Squamous cell carcinoma
SLP: Speech language pathology
WHO: World Health Organization
