# Supplementary material for: Preservation of swallowing in resected oral cavity squamous cell carcinoma: examining radiation volume effects (PRESERVE): study protocol for a randomized phase II trial
Source: Radiat Oncol. 2020 Aug 14;15:196. doi: 10.1186/s13014-020-01636-x (PMC7427897; doi:10.1186/s13014-020-01636-x)
Supplement: Supplementary file 1 — Additional file 1. Dose constraints, OAR definitions, planning priorities. [file 13014_2020_1636_MOESM1_ESM.docx]

**Additional file 1: Dose Constraints, OAR DEFINITIONS, PLANNING PRIORITIES**

Dose Constraints

Target dose constraints are shown in Supplemental Table 1, adapted from RTOG protocols 1016^36^ (Arm 1) and 0920^37^ (Arm 2), the NCIC-CTG HN6 protocol^45^, ORATOR^38,39^ and ORATOR2^40^.

Supplemental Table 1.

| Structure | Maximum dose | Planning Priority |
| --- | --- | --- |
| Organs at Risk | | |
| Spinal Cord | 48 Gy point dose  45 Gy to 0.1 cc | 1 |
| Spinal Cord PRV  (defined as spinal cord + 3-5 mm) | 52 Gy to 0.1 cc |  |
| Brainstem | 54 Gy point dose  50 Gy to 0.1 cc | 2 |
| Plexus | Maximum < 63 Gy | 6 |
| Parotid* | Mean < 26 Gy | 7 (if not in PTV) |
| Pharyngeal constrictors | Mean < 45Gy | 8 |
| Submandibular gland | Mean < 26 Gy | 9 (if not in PTV) |
| Larynx* | Maximum < 45 Gy | 10 |
| Oral Cavity* | Mean < 30 Gy | 11 |
| Lips* | Mean < 20 Gy | 12 |
| Mandible* | Maximum < 66 Gy | 13 |
| Target volumes | | |
| PTV 64 |  | 3 |
| PTV60 |  | 4 |
| PTV 54 (if present) |  | 5 |

*Maximum doses will be exceeded if the PTV overlaps with, or is in close proximity to, these structures.

Contouring definitions

**Spinal cord:** from cranial-cervical junction to T3/4. A planning organ at risk volume (PRV) will be defined as the spinal cord + 5 mm in all directions.

**Brainstem:** from the top of the midbrain to the cranial-cervical junction.

**Lips:** each lip should be contoured as a 2 cm structure in the cranio-caudal direction, and extend laterally to the commissures

**Oral cavity:** the anterior 2/3 of the tongue, floor of mouth, the buccal mucosa, and palate

**Parotid glands:** to be contoured bilaterally including the accessory lobes, not to overlap with the CTVs

**Mandible:** entire bony mandible superiorly to include coronoid and condylar processes, including the alveolar process, but excluding the teeth

**Larynx:** a triangular volume extending from the inferior aspect of the hyoid to the superior aspect of the cricoid, anteriorly to include the anterior commissure and posteriorly to include the arytenoids. It does not include the suprahyoid epiglottis.

**Pharyngeal constrictors:** a 3 mm thick strip extending from the caudal tip of the pterygoid plates to the caudal border of the cricoid cartilage.

**Contralateral Submandibular Gland:** if present, this should be contoured as the entire submandibular gland in the region of level 1B.
