# Supplementary material for: Preservation of swallowing in resected oral cavity squamous cell carcinoma: examining radiation volume effects (PRESERVE): study protocol for a randomized phase II trial
Source: Radiat Oncol. 2020 Aug 14;15:196. doi: 10.1186/s13014-020-01636-x (PMC7427897; doi:10.1186/s13014-020-01636-x)
Supplement: Supplementary file 2 — Additional file 2. Follow-up evaluation schedule. [file 13014_2020_1636_MOESM2_ESM.docx]

# Additional file 2: Follow-up Evaluation Schedule

Supplemental Table 2.

Day 1 of follow-up will be counted as the first day of radiotherapy. Additional imaging or laboratory investigations should be carried out at the discretion of the oncologist based on findings in the history or physical. Enrolment is listed as time “-t1” since allocation occurs after contours have been completed and peer-reviewed.

| **TIMEPOINT** | | | | | | | | | | | | | | | | | | | | | | |
| --- | --- | --- | --- | --- | --- | --- | --- | --- | --- | --- | --- | --- | --- | --- | --- | --- | --- | --- | --- | --- | --- | --- |
|  | **Enrolment** | **Allocation** | **Post-Allocation** | | | | | | | | | | | | | | | | | | | |
|  |  |  | **Week from Treatment Start** | | | | | | | **Month from Treatment Start** | | | | | | | | | | | | |
|  | -t1 | 0 | 1 | 2 | 3 | 4 | 5 | 6 | 12 ± 2 | 6 | 9 | 12 | 15 | 18 | 21 | 24 | 30 | 36 | 42 | 48 | 54 | 60  (close-out) |
| **ENROLMENT:** | | | | | | | | | | | | | | | | | | | | | | |
| Eligibility Screen | X |  |  |  |  |  |  |  |  |  |  |  |  |  |  |  |  |  |  |  |  |  |
| Informed Consent | X |  |  |  |  |  |  |  |  |  |  |  |  |  |  |  |  |  |  |  |  |  |
| Allocation |  | X |  |  |  |  |  |  |  |  |  |  |  |  |  |  |  |  |  |  |  |  |
| **INTERVENTIONS:** | | | | | | | | | | | | | | | | | | | | | | |
| Standard Volume RT |  |  |  |  |  |  |  |  |  |  |  |  |  |  |  |  |  |  |  |  |  |  |
| Omission of pN0 neck RT |  |  |  |  |  |  |  |  |  |  |  |  |  |  |  |  |  |  |  |  |  |  |
| **ASSESSMENTS:** | | | | | | | | | | | | | | | | | | | | | | |
| History and Physical Examination | X |  | X^1^ | X^1^ | X^1^ | X^1^ | X^1^ | X^1^ | X^1^ | X | X | X | X | X | X | X | X | X | X | X | X | X |
| CT or MRI HN | X |  |  |  |  |  |  |  | X^2^ | X^2^ |  | X^2^ |  | X^2^ |  | X^2^ |  |  |  |  |  |  |
| CT thorax or PET | X |  |  |  |  |  |  |  |  |  |  | X^3^ |  |  |  |  |  |  |  |  |  |  |
| Dental evaluation | X |  |  |  |  |  |  |  |  |  |  |  |  |  |  |  |  |  |  |  |  |  |
| FOIS score, and Modified Barium Swallow for DIGEST and MBSImP scoring | X |  |  |  |  |  |  |  |  |  |  | X |  |  |  |  |  |  |  |  |  |  |
| CTC-AE Toxicity Assessment | X |  | X | X | X | X | X | X | X | X | X | X | X | X | X | X | X | X | X | X | X | X |
| QoL Questionnaires^4^ | X |  |  |  |  |  |  | X |  | X |  | X |  | X |  | X | X | X | X | X | X | X |

^1^Laryngopharyngoscopy optional;

^2^ optional;

^3^ Chest x-ray instead of CT thorax accepted at 12 month follow-up.

^4^ Quality of Life forms include: MDADI EORTC QLQ-C30 and H&N 35, EQ-5D-5L NDII
