# Supplementary material for: Preservation of swallowing in resected oral cavity squamous cell carcinoma: examining radiation volume effects (PRESERVE): study protocol for a randomized phase II trial
Source: Radiat Oncol. 2020 Aug 14;15:196. doi: 10.1186/s13014-020-01636-x (PMC7427897; doi:10.1186/s13014-020-01636-x)
Supplement: Supplementary file 3 — Additional file 3. Study information and consent form. [file 13014_2020_1636_MOESM3_ESM.docx]

**Additional file 3: Study information and CONSENT FORM**

**Comparing normal radiation treatment areas to reduced radiation treatment areas in head and neck cancer**

Preservation of Swallowing in Resected Oral Cavity Squamous Cell Carcinoma: Examining Radiation Volume Effects (PRESERVE): A Randomized Trial

Study Doctor: Dr. ________________

Sponsor: Dr. David Palma, Lawson Health Research Institute

*If an REB approved French consent is not used at your institution remove this statement.*

Le formulaire de consentement est disponible en français sur demande.

**Emergency Contact Number** (24 hours / 7 days a week): _________________________

Non-Emergency contact numbers are at the end of this document under Contacts.

**Introduction**

You are being invited to participate in a clinical trial (a type of study that involves research). Clinical trials only include participants who choose to take part. You are invited to participate in this trial because you have had surgery for cancer of the mouth to remove the main tumour and some lymph nodes from the neck. At the time of surgery you were found to have some additional risk factors that suggest you will benefit from additional treatment with radiation therapy. For some patients it will also be recommended that you have chemotherapy with radiation. Based on results from previous studies, it has been suggested that giving radiation to a smaller treatment area may help reduce side effects without increasing the risk of the cancer coming back. This consent form provides you with information to help you make an informed choice. Please read this document carefully and take your time in making your decision. You may find it helpful to discuss it with your friends and family.

Taking part in this study is voluntary. You may choose not to take part or if you choose to participate may leave the study at any time without giving a reason. Deciding not to take part or deciding to leave the study later will not result in any penalty or any loss of benefits to which you are entitled.

**Background**

The standard or usual treatment for your disease is to administer radiation to the entire area the surgeon operated on, including the site of the tumor, the side(s) of the neck from which lymph nodes were removed, and sometimes nearby areas of the neck where lymph nodes were not taken out. Some patients may also be recommended to receive chemotherapy with radiation.

Radiation treatment to the head and neck has many potential side effects, including painful sores in the mouth and throat, irritation and sunburn of the skin, weight loss, difficulty swallowing that may require long-term use of a feeding tube, permanent mouth dryness or taste changes, hoarse voice, and thick saliva. Based on results from previous studies, it has been suggested that giving radiation to a smaller treatment area may help reduce these side effects, without increasing the risk of the cancer coming back.

This study will compare radiation therapy to the standard areas (where the surgeon operated and the neck) vs. radiation therapy to a reduced region where the side(s) of the neck that did not contain any cancer won’t receive radiation. This study aims to determine if treatment side effects will be reduced by making the area treated with radiation smaller.

The research ethics board, which oversees the ethical conduct of research involving humans, has reviewed and accepted this study.

**Purpose**

The purpose of this study is to compare the usual treatment area of radiation to a reduced treatment area to see if radiation to a smaller area on the neck reduces side effects and compare the effectiveness of the two radiation options.

**Alternative Treatments**

You do not have to take part in this study in order to receive treatment/care. Other options (in addition to the standard or usual treatment described above) may include, but are not limited to:

- no therapy at this time
- palliative care or Best Supportive Care (BSC). This type of care helps reduce pain, tiredness, appetite problems and other problems caused by the cancer. It does not treat the cancer directly, but instead tries to improve how you feel. Best Supportive Care tries to keep you as active and comfortable as possible.
- other experimental studies may be available if you do not take part in this study.

Please talk to your study doctor or usual cancer doctor about the known benefits and risks of these other options before you decide to take part in this study. Your usual cancer doctor can also discuss with you what will happen if you decide not to undertake any treatment at this time.

**Expected Number of Participants**

About 90 people will take part in this study from Canada and Scotland. This study should take 8 years to complete and the results should be known in about 9 years.

Your study doctor will be informed of the results of this study once they are known.

**Assignment to a Group**

If you decide to participate then you will be "randomized" into one of the groups described below. Randomization means that you are put into a group by chance like flipping a coin/rolling dice. There is no way to predict which group you will be assigned to. You will have a 1 in 3 chance of being placed in the ‘non-experimental treatment’ arm and a 2 in 3 chance of being placed in the ‘experimental arm.’ Neither you nor your study doctor can choose what group you will be in.

You will be told which treatment you are to get.

**Group 1 (Non-Experimental Treatment):**

If you are randomized to Group 1 you will receive standard radiotherapy to the area the tumor was removed from and both sides of the neck where the surgeon has removed lymph nodes. This will be administered every day of the week (Monday – Friday) for 6 weeks. You may also be offered standard chemotherapy (Cisplatin or Carboplatin) at a schedule determined by your doctor.

**Group 2 (Experimental Treatment):**

If you are randomized to Group 2 you will receive radiotherapy to the area the tumor was removed from and only those areas of the neck where lymph node(s) that were removed by surgery tested positive for cancer. This will be administered every day of the week (Monday – Friday) for 6 weeks. You may also be offered standard chemotherapy (Cisplatin or Carboplatin) at a schedule determined by your doctor.

**Study Procedures**

**Non-Experimental Procedures**

The following tests will be done as part of this study. Some of these tests may be done as part of your standard care, in which case the results may be used. Some of these tests may be done more frequently than if you were not taking part in this study and some may be done solely for the purpose of the study. If the results show that you are not able to continue participating, your study doctor will let you know:

- physical examination, including a laryngopharyngoscopy (looking at the back of the throat using a small camera inserted in the nose)
- speech language pathology assessment
- pregnancy test
- magnetic resonance imaging (MRI) *–* a scan that uses a strong magnet to produce pictures of areas inside the body such as organs and other tissue, and inside of bones*.* MRI scans often involve injecting a dye into your vein
- computed tomography (CT) scan – a series of x-rays of the body from many angles that are turned into 3-dimensional pictures on a screen. CT scans often involve injecting a dye into your vein.
- positron emission tomography (PET ) – a scan to help show how organs and tissues are working by tracing where a small amount of glucose (a sugar) that includes a tiny, harmless amount of radioactivity, goes in your body after it has been injected into one of your veins.
- Dental evaluation
- Modified Barium Swallow– a specialized x-ray procedure that is completed by a speech language pathologist and radiologist to examine swallowing. The speech-language pathologist will ask you to swallow different foods and liquids mixed with a contrast material called barium, and will assess your swallowing abilities.

*If applicable:* ***for centres*** *for which certain treatments, tests or procedures (e.g., scans) may take place at another location* ***please include the following information*** *with applicable modifications*

The following *treatments/procedures/* *tests* for this study may take place *closer to your home/at another location*. [*name of centre/location to be entered if applicable*]

The *information/results from these* *treatments/tprocedures/tests* will be sent to your study doctor.

- *List the treatments/ tests procedures that are authorized to take place at the above location/centre*

**Questionnaires**

You will be provided with 5 questionnaires before starting this study, and then every 6 months for 5 years while on study. The purpose of the questionnaires is to understand how your treatment and illness affects your quality of life. These questionnaires will take about 20 minutes to complete.

The information you provide is for research purposes only and will remain strictly confidential.

Some of the questions are personal; you may choose not to answer these if you wish.

Even though you may have provided information on a questionnaire, these responses will not be reviewed by your health care team or study team. If you wish them to know this information please bring it to their attention.

**SUMMARY OF STUDY PROCEDURES – Groups 1 and 2**

**Pre-Treatment (Screening) Tests**

| **Day** | **Tests, Procedures and Treatments** |
| --- | --- |
| Before study entry | - History and physical examination including laryngopharyngoscopy (looking at the back of the throat using a small camera inserted in the nose) - Assessment of your symptoms - Imaging with CT head, CT or MRI of the neck and CT chest or whole body PET-CT - Pregnancy test - Dental Evaluation - Completion of Quality of Life Forms - Modified Barium Swallow |

**Study Treatment**

| **Day** | **Tests, Procedures and Treatments** |
| --- | --- |
| 5 days a week (Monday to Friday) for 6 weeks  (1 hour) | - Radiation Therapy Treatment, with or without chemotherapy |

**During Treatment Evaluations**

| **Day** | **Tests, Procedures and Treatments** |
| --- | --- |
| Weekly during Radiation Therapy (RT) (1 hour) | - History and physical examination   to monitor any side effects you may be experiencing as a result of the treatment. |
| Week 6 of treatment  (20 minutes) | - Completion of Quality of Life Questionnaires |

**Post-Treatment Follow Up Evaluations**

|  | **Tests, Procedures and Treatments** |
| --- | --- |
| Every 3 months for first 2 years then every 6 months until 5 years (1-2 hours) | - History and physical examination, possibly including laryngopharyngoscopy (looking at the back of the throat using a small camera inserted in the nose) - Assessment of side effects you may be experiencing |
| Every 6 months until 5 years  (20 minutes) | - Completion of Quality of Life Questionnaires |
| 12 month follow up visit | - Modified Barium Swallow - Imaging with CT of the head and neck and chest |
| 6 weeks post radiation, month 6, 12, 18 and 24 | - Imaging with CT head and neck |

**Responsibilities**

If you choose to participate in this study, you will be expected to:

- Tell your study doctor about your current medical conditions;
- Tell your study doctor if you are thinking about participating on another research study;
- Tell your study doctor about all prescription and non-prescription medications and supplements, including vitamins and herbals, and check with your study doctor before starting, stopping or changing any of these. This is for your safety as these may interact with the treatment you receive on this study;
- Return any questionnaires that were completed to the clinic/hospital;
- Tell your study doctor if you become pregnant or father a child while participating on this study

**Length of Participation**

Your radiation treatment will last for 6 weeks.

After your treatment ends, you will be asked to come back to the clinic/hospital every 3 months for 2 years and then every 6 months until 5 years. You may be seen more often if your study doctor determines that this is necessary, or if your cancer returns.

No matter which group you are randomized to, and even if you stop treatment early, the researchers would like to keep track of your health for 5 years to look at the long-term effects of your participation on this study. This would be done at your usual follow up visits and asking your doctor about your health, or by looking at your medical records to check your health status. You will also be asked to complete the follow up modified barium swallow test, imaging tests and quality of life forms according to the follow up schedule above.

**Early End to Participation**

Your participation in the trial may be stopped early, for reasons such as:

- The treatment does not work for you and your cancer comes back
- You are unable to tolerate the study treatment.
- You are unable to complete all required study procedures.
- New information shows that the study treatment is no longer in your best interest.
- Your study doctor no longer feels this is the best treatment for you.
- The sponsor decides to stop the study.
- The Research Ethics Board withdraws permission for the study to continue.
- If you become pregnant.

If your participation in the study is stopped your study doctor will provide information about how to stop safely.

**Risks of Participation**

You will be receiving standard radiation therapy with or without chemotherapy in this study.

The risks and side-effects of the standard or usual treatment will be explained to you as part of your standard care and are therefore not listed.

This study attempts to reduce the area treated by radiation. The chances that cancer could come back in the side of the neck where treatment was omitted may be slightly higher compared to standard treatment. Cancer recurrence may mean that you may need additional treatment (surgery, radiation or chemotherapy), and may affect your chances of being permanently cured of cancer.

Your physician will monitor your therapy and make adjustments to your treatment or prescribe medicines in order to manage side effects that occur during treatment. You will be closely followed after treatment to monitor for cancer recurrence.

**Reproductive Risks**

The effects that radiation may have on an unborn baby (fetus) are unknown. You must not become pregnant or father a baby while taking part in this study and for 6 months after the last dose of radiation or chemotherapy.

Your study doctor will discuss methods with you to ensure that you do not become pregnant or father a baby during the study.

Women should not nurse (breastfeed) a baby during the study treatment period and for 6 months after the last dose because the radiation used in this study might be present in breast milk and could be harmful to a baby.

**Data Safety Monitoring Board/Committee**

A Data Safety Monitoring Board/Committee, an independent group of experts, will be reviewing the data from this research throughout the study.

**Benefits**

If you agree to take part in this study, the experimental treatment may or may not be of direct benefit to you. It is possible that the experimental treatment will reduce the side effects of radiation treatment, including less difficulty with swallowing, dry mouth and taste changes, however this is still unknown.

The researchers hope the information learned from this study will help other patients in the future.

**Confidentiality**

Records identifying you at this centre will be kept confidential and, to the extent permitted by the applicable laws, will not be disclosed or made publicly available, except as described in this consent document.

Authorized representatives of the following organizations may look at your original (identifiable) medical/clinical study records at the site where these records are held, for quality assurance (to check that the information collected for the study is correct and follows proper laws and guidelines):

- Dr. David Palma, the sponsor of this study or his delegate
- The Ontario Cancer Research Ethics Board, which oversees the ethical conduct of this study in your clinic/hospital;

Authorized representatives of the above organizations and the organization listed below may **receive** information related to the study from your medical/clinical study records for quality assurance and data analysis. Your name or other information that may identify you will not be used. The records received by these organizations may contain your participant code, initials, sex and partial date of birth.

- The study central review office at the London Regional Cancer Program

All of the organizations listed in the above confidentiality sections are required to have strict policies and procedures to keep the information they see or receive about you confidential, except where disclosure may be required by law. The study doctor will ensure that any personal health information collected for this study is kept in a secure and confidential location as required by law. There are federal and provincial laws that these organizations must comply with to protect your privacy.

If the results of this study are published, your identity will remain confidential. It is expected that the information collected during this study will be used in analyses and will be published/ presented to the scientific community at meetings and in journals.

Even though the likelihood that someone may identify you from the study data is very small, it can never be completely eliminated.

A copy of this signed and dated consent form may be included in your health record/hospital chart.

Your family doctor/health care provider will be informed that you are taking part in a study so that you can be provided with appropriate medical care. If you do not want your family doctor/health care provider to be informed, please discuss with your study doctor.

A wallet card will be provided to you with information about how to contact the study staff when required

- Your de-identified data from this study may be used for other research studies. If your study data is shared with other researchers, information that links your study data directly to you will not be shared.

**Registration of Clinical Trials**

A description of this clinical trial will be available on <http://www.clinicaltrials.gov>. This Web site will not include information that can identify you. At most, the Web site will include a summary of the results. You can search this Web site at any time.

**Costs**

The costs of your medical treatment will be paid for by your provincial medical plan to the extent that such coverage is available. There may be extra costs that are not covered by your medical plan that you will have to pay yourself; some examples may be physiotherapy or certain pain medications.

Taking part in this study may result in added costs to you (i.e. transportation, parking, meals, or unpaid leave from work). You may have to pay for medication prescribed to treat or prevent side effects, and you may have to visit the hospital more often than if you were not participating in this study.

**Compensation**

You will not be paid for taking part in this study.

*You will be reimbursed for study-related expenses such as specify, e.g., parking, etc.*

*Note: this statement may be removed/revised as per centre requirements.*

It is possible that the research conducted using your study data may eventually lead to the development of new diagnostic tests, new drugs or other commercial products. There are no plans to provide payment to you if this happens.

In the case of research-related side effects or injury, medical care will be provided by your doctor or you will be referred for appropriate medical care.

**Rights**

You will be told, in a timely manner, about new information that may be relevant to your willingness to stay in this study.

If you decide to stop participating in the study or if your participation has been stopped, your doctor will discuss other options with you and continue to treat you with the best means available.

You may withdraw your permission to use your personal health information for this study at any time by letting the study doctor know. However, this would also mean that you withdraw from the study. Your study data that was recorded before you withdrew will be used but no information will be collected or sent to the sponsor after you withdraw your permission.

Your rights to privacy are legally protected by federal and provincial laws that require safeguards to ensure that your privacy is respected.

By signing this form you do not give up any of your legal rights against the investigators, sponsor or involved institutions for compensation, nor does this form relieve the investigators, sponsor or involved institutions of their legal and professional responsibilities.

You will be given a copy of this signed and dated consent form prior to participating in this study.

**Conflict of Interest**

This centre is receiving funds from the study Sponsor, from a philanthropic donation and from a study grant from the Ontario Institute for Cancer Research through funding provided by the Government of Ontario, to help offset the costs of conducting this research. The researchers at this centre will not receive any direct benefit for conducting this study.

The doctor treating you also may be the doctor in charge of the study.

If you would like additional information about the funding for this study, or about the role of the doctor in charge of this study, please speak to the study staff or to the Office of the Chair of the Ontario Cancer Research Ethics Board. (contact information below)

**Contacts**

If you have questions about taking part in this study, or if you suffer a research-related injury, you should talk to your study doctor. Or, you can meet with the doctor who is in charge of the study at this institution. That person is:

| *Name* |  | *Telephone #* |
| --- | --- | --- |

If you have questions about your rights as a participant or about ethical issues related to this study, you can talk to someone who is not involved in the study at all. Please contact the Office of the Chair of the Ontario Cancer Research Ethics Board at:

| Telephone: 416-673-6648 |  | Toll Free: 1-866-678-6427 ext. 6648 |
| --- | --- | --- |

**Signatures**

- All of my questions have been answered,
- I understand the information within this informed consent form,
- I allow access to my medical records and specimens as explained in this consent form,
- I am aware of the risks to me of participating in the study and the risks to the fetus if I become pregnant or father a child during this study,
- I do not give up any of my legal rights by signing this consent form,
- I agree to take part in this study.

| Signature of Participant |  | Printed Name |  | Date |
| --- | --- | --- | --- | --- |

| Signature of Person Conducting the Consent Discussion |  | Printed Name |  | Date |
| --- | --- | --- | --- | --- |

**Participant Assistance**

**Complete the following declaration only if the participant is unable to read:**

- The informed consent form was accurately explained to, and apparently understood by, the participant, and
- Informed consent was freely given by the participant

| Signature of Impartial Witness |  | Printed Name |  | Date |
| --- | --- | --- | --- | --- |

**Complete the following declaration only if the participant has limited proficiency in the language in which the consent form is written and interpretation was provided as follows:**

- The informed consent discussion was interpreted by an interpreter, and
- A sight translation of this document was provided by the interpreter as directed by the research staff conducting the consent.

**Interpreter Declaration and Signature:**

By signing the consent form I attest that I provided a faithful interpretation for any discussion that took place in my presence, and provided a sight translation of this document as directed by the research staff conducting the consent.

| Signature of Interpreter |  | Printed Name |  | Date |
| --- | --- | --- | --- | --- |
