# Supplementary material for: Preservation of swallowing in resected oral cavity squamous cell carcinoma: examining radiation volume effects (PRESERVE): study protocol for a randomized phase II trial
Source: Radiat Oncol. 2020 Aug 14;15:196. doi: 10.1186/s13014-020-01636-x (PMC7427897; doi:10.1186/s13014-020-01636-x)
Supplement: Supplementary file 4 — Additional file 4. WHO trial registration data set. [file 13014_2020_1636_MOESM4_ESM.docx]

**Additional file 4: WHO Trial Registration Data Set**

| **Item** | **Description** |
| --- | --- |
| Primary registry and trial identifying number | ClinicalTrials.gov : NCT03997643 |
| Date of registration in primary registry | June 25, 2019 |
| Secondary identifying numbers | NA |
| Source(s) of monetary or material support | London Health Sciences Foundation (philanthropic donations) and Ontario Institute of Cancer Research |
| Primary sponsor | Lawson Health Research Institute |
| Secondary sponsor(s) | NA |
| Contact for public queries | Dr. David A. Palma |
| Contact for scientific queries | Dr. David A. Palma |
| Public title | Preservation of Swallowing in Resected Oral Cavity Squamous Cell Carcinoma: Examining Radiation Volume Effects: A Randomized Trial |
| Scientific title | Preservation of Swallowing in Resected Oral Cavity Squamous Cell Carcinoma: Examining Radiation Volume Effects: A Randomized Trial |
| Countries of recruitment | Canada, Scotland, United States |
| Health condition(s) or problem(s) studied | Squamous cell carcinoma of the oral cavity |
| Intervention(s) | Standard radiation volumes |
|  | Omission of radiation to the pathologically node negative dissected neck |
| Key inclusion and exclusion criteria | Inclusion: Age 18 or older  Willing to provide informed consent  ECOG performance status 0-2  Resected OCSCC with at least an ipsilateral selective neck dissection. The oral cavity includes: lips, buccal mucosa, oral tongue, floor of mouth, gingiva, retromolar trigone, and hard palate.  Patient has at least one pathological feature that is an indication for PORT: positive or close (≤ 3 mm) margin, presence of LVI or PNI, pT3 or pT4 disease, positive lymph nodes, or extranodal extension  PORT is recommended by the treating physician  Pathologically lymph node negative in at least one dissected hemi-neck with at least 10 nodes recovered in each pN0 hemi-neck, after a dissection that at minimum includes nodal levels 1-3 in the pN0 hemi-neck(s).  Radiation contours have been peer-reviewed and approved |
|  | Exclusion:  Patients with an ipsilateral neck dissection only with positive lymph nodes, unless they undergo a contralateral neck dissection that is pN0  Patients with bilaterally involved neck nodes  Serious medical comorbidities or other contraindications to radiotherapy  Prior history of head and neck cancer within 5 years  Any other active invasive malignancy, except non-melanotic skin cancers.  Prior head and neck radiation at any time  Prior oncologic head and neck surgery in the oral cavity or neck.  Known metastatic disease  Locoregional disease recurrence identified following surgical resection but prior to start of radiotherapy  Inability to attend full course of radiotherapy or follow-up visits  Unable or unwilling to complete QoL questionnaires  Pregnant or lactating women |
| Study type | Randomized by permuted blocks sequence |
|  | No masking/blinding (open label) |
|  | Parallel assignment |
| Date of first enrolment | December 16, 2019 |
| Target sample size | 90 |
| Recruitment status | Recruiting |
| Primary outcome(s) | Regional failure in the pN0 hemi-neck(s) |
| Key secondary outcomes | Quality of life, assessed with the MD Anderson Dysphagia Inventory (MDADI), the EORTC QLQ-C30 and H&N35 scales, the EQ-5D-5L, and the neck dissection impairment index (NDII).  Overall survival  Disease-free survival  Local recurrence  Regional recurrence  Locoregional recurrence  Rate of salvage treatment (surgery +/- radiotherapy) in the pN0 neck, and freedom from unsalvagable neck recurrence  Rate of feeding tube insertion after start of radiation (either gastric, gastrojejeunal, or nasogastric) and rate of feeding tube use at 1-year post-randomization.  Swallowing function at 1-year, measured by the Modified Barium Swallow Impairment (MBSimp) score, the Dynamic Imaging Grade of Swallowing Toxicity (DIGEST) score, and the Functional Oral Intake Score (FOIS).  Toxicity, assessed using the National Cancer Institute Common Toxicity Criteria (NCI-CTC) version 4.03  Rate of failure in the clinically node negative neck, if applicable (i.e. the undissected node-negative neck, for well-lateralized tumors). |
